# Editorial: Preserving health: health technology for fall prevention

**DOI:** 10.3389/fdgth.2024.1530434

**Published:** 2024-11-29

**Authors:** Thurmon Lockhart

**Affiliations:** Locomotion Research Lab, School of Biological and Health Systems Engineering, Arizona State University, Tempe, AZ, United States

**Keywords:** fall risk assessment, gait and posture, ADL (activities of daily life), osteoporosis, multiple sclerosis, older adults, machine learning algorithms, mobile technology

**Editorial on the Research Topic**
Preserving health: health technology for fall prevention

Market projections for health monitoring sensors and network technologies are anticipated to grow significantly in coming years. The emergence of mobile health monitoring systems in the healthcare sector is currently driving a transformation that includes a shift from treatment of disease to prevention, a personalization of medical care and a shift of medical standards based on continuous physiological data obtained from a large body of population. Motivated by healthcare cost increase and driven by recent technological advances in miniature electromechanical devices, microelectronics, and wireless communications, the health monitoring sensing technology sector is growing rapidly and has the potential to transform the future of healthcare. In this special issue, we present a new fall risk assessment paradigm utilizing mobile and wearable technology that can transform fall risk assessment technology.

Falls and gait instability are among the most serious problems facing older adults and constitute a major cause of mortality, morbidity, immobility, and premature nursing home placement. Fall risk assessment can be a powerful tool for early diagnosis and treatment of falls if done correctly. However, mobility impairment and associated fall risk which can be subtle is often missed, further increasing the risk of a fall. Early detection of impaired mobility and increased fall risk are thus critical to timely interventions prior to falling episodes.

In recent years, many technologies for mobility, balance, and fall detection in various populations have emerged. Wearable sensors, passive in-house monitors, and many combinations thereof all promise to alert caregivers or emergency personnel once a fall is detected. But for many individuals, detecting a fall once it has occurred is already too late—the damage has been done, outcomes are typically poor, and cost is always high. What is needed for health preservation is fall prevention. While falls are a major problem that is growing as the population is rapidly ageing, most of the current technology and care solutions are based on a one-dimensional approach. However, the causes leading to falls are varied and are better understood by accessing information at different scales and populations.

To our knowledge, there has been no previous effort to address fall prevention from multiple levels of monitoring and predictive perspectives. In this Research Topic, we present a few studies by researchers who are at the helm of concurrently measuring multiple functions among older adults as well as those with pathology. Especially those involved in fall risk assessments and fall risk prediction areas, including balance and mobility.

Although biomechanical and physiological parameters associated with mobility issues and fall risk have been established by testing/collecting cohort relevant data at the population levels, currently, it is unknown, how these features can be used for personalized assessments in international environments. The fundamental contribution of this Research Topic is to provide cogent features/models relevant for health assessments utilizing multimodal, time varying physiological and biomechanical fall risk characteristics utilizing a variety of subjective and objective techniques.

“*The Developments and Iterations of a Mobile Technology-Based Fall Risk Health Application*,” by Hsieh et al., from the Department of Internal Medicine, Section on Gerontology and Geriatric Medicine, Wake Forest School of Medicine, Winston-Salem, NC, United States, Department of Kinesiology and Community Health, University of Illinois at Urbana-Champaign, Champaign, IL, United States, Department of Health and Exercise Science, Wake Forest University, Winston-Salem, NC, United States, and Department of Physical Therapy, Rehabilitation Science, and Athletic Training, University of Kansas Medical Center, Kansas City, KS, United States.

In this study, Authors introduced a Mobile Technology-Based Fall Risk Health Application that can assess fall risk of special populations' fall risk—e.g., individuals with Multiple Sclerosis (MS) and Wheeled device users. Authors assert that mobile technology can be leveraged to provide personalized fall risk screening for different clinical populations. Additionally, they indicated that fall risk applications should be designed to tailor one's specific stability weaknesses to be measured and intervened.

“*Explainable Fall Risk Prediction in Older Adults Using Gait and Geriatric Assessment*,” by Mishra et al., from the Department of Electrical Engineering and Computer Science, University of Missouri, Columbia, MO, United States, Sinclair School of Nursing, University of Missouri, Columbia, MO, United States, Department of Health Management and Informatics, University of Missouri, Columbia, MO, United States, School of Health Professions, Physical Therapy, University of Missouri, Columbia, MO, United States, Department of Health Sciences Research, Mayo Clinic, Rochester, MN, United States, Department of Gastroenterology and Hepatology, Mayo Clinic, Rochester, MN, United States.

This manuscript describes use of gait parameters (the Functional Ambulatory Profile scores and gait speed) from GAITRite system and fall history of 92 older adults to create a fall risk prediction system using a machine learning model that may be able explain the fall risk for individuals and, thereby knowing how to effectively intervene as the model will provide those variables of interest. Currently, machine learning in healthcare domain usually uses statistical parameters (e.g., zero crossings, skewness, etc.) that may not be well suited to delivering effective interventions or outline fall risk related to individual needs. As such, explainable machine learning is very important for fall risk prediction and fall prevention as the models will provide what to work on to reduce the risk of falls.

“*Wearable Sensor Systems for Fall Risk Assessment: A Review*,” by Subramaniam et al., from the School of Biomedical Engineering, McMaster University, Hamilton, ON, Canada, and Electrical and Computer Engineering, McMaster University, Hamilton, ON, Canada.

In this manuscript, the authors provide a cogent review of fall risk assessment technology based on inertial measurement units (IMUs) and insole device to capture gait parameters and center-of-pressure information in order to assess postural and gait stability to ascertain fall risk of an individual. Critical measures of gait stability to include in the design of these types of system is elaborated further. In summary, the review identifies key points including spatiotemporal parameters, biomechanical gait parameters, physical activities and data analysis methods pertaining to recently developed systems, current challenges, and future perspectives.

In “*Assessing fall risk in osteoporosis patients: a comparative study of age-matched fallers and nonfallers*,” by Hyun Moon et al., from the Locomotion Research Laboratory, School of Biological and Health Systems Engineering, Arizona State University, Tempe, AZ, United States and Division of Endocrinology, Mayo Clinic, Scottsdale, AZ, United States.

In this study, the authors tested fallers and non-fallers (all with osteoporosis) gait and postural stability characteristics to assess fall risk at the clinic as well as at their own homes. Furthermore, various biomarkers were collected on these participants to further relate to fall risk. The study indicated that fall risk can be assessed at their home using IMU and converting it into energy (or frequencies of activities) and found that fallers were less active than their counterparts. Additionally, some biomarkers, such as low levels of calcium and Parathyroid hormone, Vitamin D, and renal function were associated with fallers. This study highlights the that biomarker as well as gait and postural stability measures can be assessed to characterize frequent fallers.

